# The Value of Structural Neuroimaging in First-Episode Psychosis and the Prevalence of Imaging Abnormalities and Clinical Relevance: A Real-World Observational Study

**DOI:** 10.3390/jcm14144925

**Published:** 2025-07-11

**Authors:** José Pablo Martínez Barbero, José Tortosa Cámara, Beatriz Ramos Barbosa, Paula María Jiménez Gutiérrez, Manuel González Díez, José Eduardo Muñoz Negro, José Prados, Antonio Jesús Láinez Ramos-Bossini

**Affiliations:** 1 Department of Radiology, Hospital Universitario Virgen de las Nieves, 18014 Granada, Spain; jpmbhg@gmail.com; 2 Instituto de Investigación Biosanitaria de Granada ibs.GRANADA, 18016 Granada, Spain; jose.tortosa.sspa@juntadeandalucia.es (J.T.C.); apaulajimenezg@gmail.com (P.M.J.G.); jcprados@ugr.es (J.P.); 3 Department of Human Anatomy and Embryology, School of Medicine, University of Granada, 18071 Granada, Spain; beatrizramos@correo.ugr.es; 4 Department of Anesthesiology, Hospital Universitario Virgen de las Nieves, 18014 Granada, Spain; 5 Department of Psychiatry, Psychotherapy and Psychosomatic Medicine, District Hospital Bayreuth Psychiatric Health Care Facilities of Upper Franconia, 95445, Bayreuth, Germany; manologonzalezdiez10@gmail.com; 6 Department of Psychiatry, School of Medicine, University of Granada, 18071 Granada, Spain; jemunoznegro@ugr.es; 7 Institute of Biopathology and Regenerative Medicine (IBIMER), University of Granada, 18100 Granada, Spain; 8 Center of Biomedical Research (CIBM), University of Granada, 18100 Granada, Spain

**Keywords:** first-episode psychosis, brief psychotic disorder, computed tomography, magnetic resonance imaging, neuroimaging

## Abstract

**Introduction**: The usefulness of neuroimaging in patients with first-episode psychosis (FEP) remains controversial. The aim of this study was to assess the prevalence and types of structural abnormalities on neuroimaging in patients with FEP and identify the most frequently used imaging modalities in a real-world setting. **Methodology:** A retrospective observational study based on a consecutive series of patients admitted to our institution with FEP was conducted. We analyzed the imaging tests performed, the presence of specific lesions, the degree of cortical atrophy (Global Cortical Atrophy, GCA scale), medial temporal atrophy (Medial Temporal lobe Atrophy, MTA scale) and non-specific white matter lesions (Fazekas scale). Descriptive and bivariate analyses were performed according to previously established age cut-offs. **Results:** A total of 105 patients were included (median age: 36 years; 52.4% men). The most frequently used neuroimaging test was computed tomography (CT) (92.4%). GCA scores that were out of the age range were found in 32.4% of patients, being more frequent in those older than 65 years (*p* < 0.001). Out-of-range MTA scores were found in 36.2% of patients, especially in patients older than 75 years (*p* < 0.001). Out-of-range Fazekas scores were found in 4.3% of patients, especially in patients older than 70 years (*p* = 0.157). Finally, only one specific structural lesion (right frontal cavernoma) was identified in one patient (1%). Overall, at least one non-age-matched abnormality was found in 46.7% of patients. **Conclusions:** Although non-specific alterations not in accordance with age exist in a significant percentage of patients with FEP, the prevalence of specific lesions is very low. This suggests that neuroimaging tests could be restricted in patients with FEP, especially CT, due to the risks associated with ionizing radiation. However, further prospective and controlled studies are needed to validate our results.

## 1. Introduction

Psychotic experiences occur frequently in the general population, with a lifetime prevalence of 5.8% [1] and an annual incidence of new psychotic episodes of 2% [2]. The clinical significance of psychosis-related conditions can vary considerably, from mild and isolated episodes to severe and recurrent psychotic disorders [3]. First-episode psychosis (FEP) refers to the first event of psychotic symptoms in a patient (e.g., delusions, hallucinations and/or disorganized thinking), regardless of the time of evolution of the symptoms [4]. This clinical presentation is very heterogeneous, as it is often accompanied by a variety of additional signs and symptoms—both psychotic and non-psychotic—that complicate its evaluation and management [5,6]. Of note, it is important to differentiate FEP from other psychiatric entities that typically require a different clinical approach, as is the case with delirium in the elderly, which often includes fluctuating levels of consciousness and cognitive impairment, or substance-induced psychosis, which may be more common in younger individuals.

Despite its clinical relevance, FEP is not included as a specific diagnostic category in the current versions of the main disease classification systems used in clinical practice, such as the International Classification of Diseases (ICD-10/11) or the Diagnostic and Statistical Manual of Mental Disorders (DSM-5) [7]. In ICD-10, FEP is usually included within a broad range of psychotic disorders grouped mainly under categories F20-F29 that include, among others, schizophrenia (F20), acute and transient psychotic disorders (F23), schizotypal disorders (F21) and delusional disorders (F22) [8]. In ICD-11, the categories for psychotic disorders have been reorganized and updated compared to the ICD-10 [9]. In particular, F23 disorders are now classified under “Acute and Transient Psychotic Disorder” with more specific criteria to differentiate them from other psychotic disorders.

The absence of a precise diagnostic category for FEP is an indicator of the lack of knowledge that still exists about its biological basis. However, it is known that various genetic, neurobiological, psychosocial and environmental factors are involved in its etiopathogenesis. In this context, routine evaluation of FEP includes, in addition to a rigorous clinical history and physical examination, the use of complementary tests, with neuroimaging examinations being relevant to rule out possible organic causes such as tumors, infections, ischemia or malformations [10,11]. However, there has been significant dispute over the role and indications of neuroimaging in the diagnosis and therapeutic approach to FEP [12,13].

Continuous improvements in medical imaging equipment in recent decades have significantly contributed to non-invasive evaluation of the brain [14]. Magnetic resonance imaging (MRI) is usually the reference test due to its high sensitivity, specificity and safety profile, and the fact that it does not use ionizing radiation [15]. However, it involves a higher cost and demand for technical resources that may not be available in various clinical settings [16]. On the other hand, computed tomography (CT) is more accessible, less expensive and faster, but carries risks related to exposure to ionizing radiation, which limits its recommendation as a first-line tool in the absence of clear indications [17]. The variability in costs of brain CT and MRI is significant across countries and conditioned by the health system, but in general, the price of MRI is twice that of CT [18].

Neuroimaging has proven useful in better understanding the pathophysiological basis of FEP. Studies on specific structures, such as the hippocampus and amygdala, have linked atrophy of these regions with the severity of psychotic symptoms [19]. Such findings support the idea that psychotic disorders are not merely functional phenomena, but may also have detectable neuroanatomical correlates. However, most studies aimed at this type of analysis have been conducted in research contexts, employing advanced structural analyses that are not used in routine clinical practice.

Regarding radiological findings identifiable in routine clinical practice, non-specific alterations such as T2 hyperintensities in the periventricular white matter and ventricular asymmetries in patients with FEP and chronic psychosis have been described [20], as well as anecdotal cases of associated arachnoid cysts [21,22,23]. Overall, the prevalence of radiological abnormalities considered clinically relevant in MRI in patients with FEP has been estimated at 6% according to a recent systematic review [24]. However, a more detailed analysis of these alterations raises reasonable doubts about their clinical significance, since they are common findings in an asymptomatic population, so their causal relationship with psychosis is questionable. In addition, other studies have reported a wide range in the prevalence variability of neuroimaging findings, ranging from 0% [12] to over 60% [25]. In most cases, these findings do not influence the subsequent clinical management of patients with FEP.

While MRI is useful to rule out other potentially treatable medical conditions, its systematic routine implementation raises questions regarding its clinical and bioethical justification, given the balance between benefits, risks and associated costs. Since MRI is safe, in this case, the dilemma centers mainly on a question of cost efficiency. However, in clinical practice, due to the lower accessibility of this technique and the occasional need to make a diagnosis in an urgent context, it is common to perform brain CT, increasing the risk of a non-justified systematic use of this examination that involves ionizing radiation.

This last scenario makes it necessary to explore in greater detail the real-world usefulness of neuroimaging tests in patients with FEP, since if these do not identify relevant radiological findings in a significant percentage of cases, their use as a routine complementary test in the approach to FEP should be reconsidered, particularly in the case of CT. On the other hand, further knowledge on the prevalence of non-specific alterations and their clinical significance is still required. The aim of this study was to determine the prevalence and types of radiological alterations in patients with FEP and the use of neuroimaging examinations in a real-world scenario. In this way, we intend to contribute to the existing body of knowledge on this topic by gathering information from a Spanish tertiary-level hospital over almost a decade.

## 2. Materials and Methods

### 2.1. Study Design and Patient Selection

A retrospective observational study was carried out based on a consecutive series of patients admitted to the Psychiatry and Mental Health Service of the Hospital Universitario Virgen de las Nieves (HUVN) from 1 January 2016 to 31 December 2024. The design and writing of this study were based on the Strengthening the Report of Observational Studies in Epidemiology (STROBE) guidelines [26]. The study protocol was approved by the Provincial Ethics Committee of Granada (code: TFG-PEP25). Given the retrospective nature of the study, written informed consent was waived.

Regarding the selection criteria, the ICD-10 diagnostic coding system is used at HUVN. Since this system does not have a specific category for FEP, in order to reduce selection bias, all ICD-10 coding (F20–F29) associated with psychotic disorders was reviewed, and cases classified as Brief Psychotic Disorder (F23) were selected. Subsequently, the medical records were reviewed to ensure that there were no previous episodes of psychosis. Inclusion and exclusion criteria are detailed below:

Inclusion criteria:
  - Patients of legal age (>18 years old).
  - Diagnosis of Brief Psychotic Disorder (F23 according to ICD-10) at discharge recorded in the minimum core data set of the clinical information system.
  - Absence of psychotic episode prior to admission according to medical history information.
  - Available structural neuroimaging test (CT and/or MRI) within 30 days before or after the date of admission.
Exclusion criteria:
  - History of brain surgery or moderate or severe traumatic brain injury.
  - Lack of neuroimaging tests available in the hospital Picture Archiving and Communication System (PACS).
  - Non-diagnostic quality of imaging tests (artifacts, incomplete study, etc.).
  - Lack of radiological report.
  - Lack of ICD-10 coding.

### 2.2. Study Variables

For each patient included in the study, we collected different data, including demographic variables (age and sex), neuroimaging tests performed (CT, MRI or both), presence of specific structural alterations, and presence and degree of non-specific abnormalities, including the degree of brain atrophy, the degree of Medial Temporal Lobe Atrophy, and the amount of non-specific white matter lesions (the latter was only evaluated for patients with available MRI).

For the assessment of non-specific alterations, we employed three scales widely used in clinical practice, as follows:

*Global Cortical Atrophy* (GCA, Table 1) [27]: This is a qualitative assessment method used to evaluate cortical brain atrophy. This method considers the evaluation of atrophy in 13 brain regions evaluated separately in each hemisphere. For each of them, a score from 0 to 3 is assigned depending on the severity of atrophy. The final score assigned corresponds to the sum of the scores for each region. The assessed regions include the following:

- Dilatation of sulci: frontal, parieto-occipital, and temporal (right and left).
- Ventricular dilatation: frontal, parieto-occipital, and temporal (right and left); third ventricle.

*Medial Temporal Lobe Atrophy Scale* (MTA, Table 2) [30]: This is used to assess the degree of cortical atrophy in the mesial region of the temporal lobe. The overall score is obtained by averaging the scores on the right and left sides. Age cut-offs have been established to consider it as pathological [31,32]:

- For individuals under 50 years of age, any score above 0 is considered atrophy.
- For individuals aged 50 to 64 years, atrophy is considered when the overall score is equal to or greater than 1.
- For individuals aged 65 to 74 years, atrophy is considered when the overall score is equal to or greater than 1.5.
- For individuals aged 75 years or older, atrophy is considered when the overall score is equal to or greater than 2.

*Fazekas Scale* (Table 3) [33,34]: This is used to quantify the number of hyperintense lesions in T2/T2 FLAIR of the white matter. The scale was originally designed to differentiate between lesions located in the periventricular white matter and in the deep white matter (corona radiata and semioval centers). However, in clinical practice, a single global value, corresponding to the highest scoring category of both white matter locations, is normally used.

### 2.3. Statistical Analysis

Univariate descriptive analyses were performed. For the quantitative variable “age”, normality analyses were carried out using the Shapiro–Wilk test and an analysis of its distribution according to sex, expressing its central distribution and main dispersion parameters. Qualitative variables were expressed as absolute and relative frequencies.

Subsequently, bivariate contrastive analyses were performed using two-tailed Fisher’s exact tests to study the association between GCA, MTA and Fazekas scores according to age ranges of the patients, establishing those corresponding to the thresholds previously described for each scale as the cut-off points. This way, we aimed to provide more specific cues on the distribution of radiological abnormalities, considering previously established thresholds.

## 3. Results

### 3.1. Characteristics of the Subjects Included in the Study

**Figure 1:**
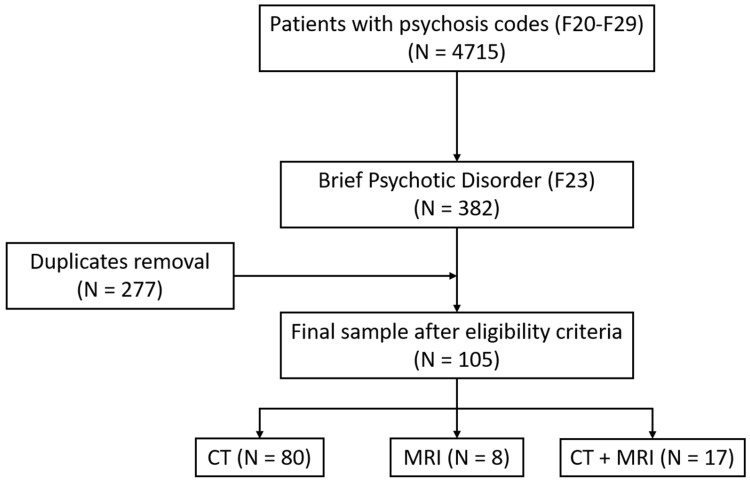
Flow chart of the patients’ selection process.

Of the 4715 patients with ICD-10 coding related to psychotic symptoms (F20–F29), 382 corresponded to Brief Psychotic Disorder (F23), which included 277 duplicated records that were removed. After applying the inclusion and exclusion criteria, the final study sample consisted of 105 patients. Figure 1 shows the flow diagram of the study.

The median age of the sample was 36 years, with a similar distribution by sex (52.4% in men). The Shapiro–Wilk test showed that the hypothesis of normality for age could not be accepted (*p* = 0.001). The analysis of this variable according to sex showed that it followed a normal distribution in women (*p* = 0.115), but not in men (*p* = 0.008). The Wilcoxon test to compare both groups showed a tendency towards significance, indicating greater age in women than in men (*p* = 0.06). Supplementary Figure S1 shows the age distribution in the sample, and Supplementary Figure S2 shows the age distribution according to sex.

A total of 80 cases (76.2%) corresponded exclusively to CT, 8 (7.6%) to MRI, and 17 (16.2%) underwent both CT and MRI during admission. Supplementary Figure S3 shows the distribution of age by neuroimaging tests used in the sample.

Considering the age ranges for which certain non-specific brain alterations (GCA, MTA and Fazekas scores) can be considered within normal range, the number of cases with any type of structural alteration was 48 (45.7%). Supplementary Figure S4 shows the distribution of cases with in-range and out-of-range non-specific abnormalities in the sample. In addition, another case without alterations in these scales presented a specific structural lesion, so the total number of cases with structural lesions was 49 (46.7%). Table 4 shows the main characteristics and findings in the sample.

### 3.2. Cortical Atrophy, Medial Temporal Lobe Atrophy and Non-Specific White Matter Lesions

#### 3.2.1. Cortical Atrophy

**Figure 2:**
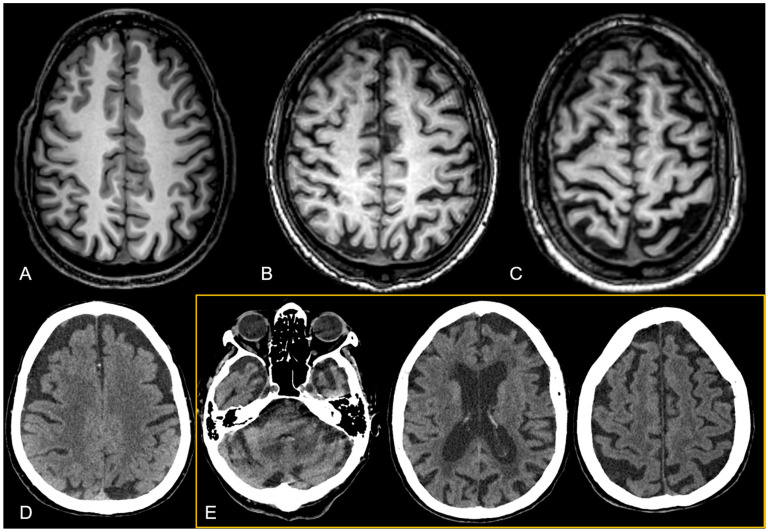
Illustrative examples of the GCA scale in magnetic resonance imaging (MRI) and computed tomography (CT) in our sample. (**A**) GCA 0 in a patient aged 30 years who presented with auditive and visual hallucinations. (**B**) GCA 1 in a 53-year-old patient who presented with visual hallucinations. (**C**) GCA 2 in a 70-year-old patient who presented with auditive hallucinations (note a slightly more pronounced sulci opening in the left hemisphere due to asynclitic horizontal disposition of the slice). (**D**) CT findings consistent with GCA 2 in an 80-year-old patient who presented with visual hallucinations (note significant bilateral frontal atrophy). (**E**) Different slices showing CT findings consistent with severe atrophy (GCA 3), more pronounced in the temporal and left frontal gyri, in a 79-year-old patient.

The GCA score was 1 in 28 patients (26.7%), 2 in 5 patients (4.8%) and 3 in 1 patient (1%). In total, 34 patients (32.4%) had an abnormal GCA score considering the age range. Fisher’s exact test showed a statistically significant association, with a higher presence of abnormal GCA for age in patients aged 65 years or older. Table 5 and Supplementary Figure S4 show the distribution of in-range and out-of-range GCA according to the age cut-off. Figure 2 shows illustrative examples of the GCA scale in patients from the sample.

#### 3.2.2. Medial Temporal Lobe Atrophy

**Figure 3:**
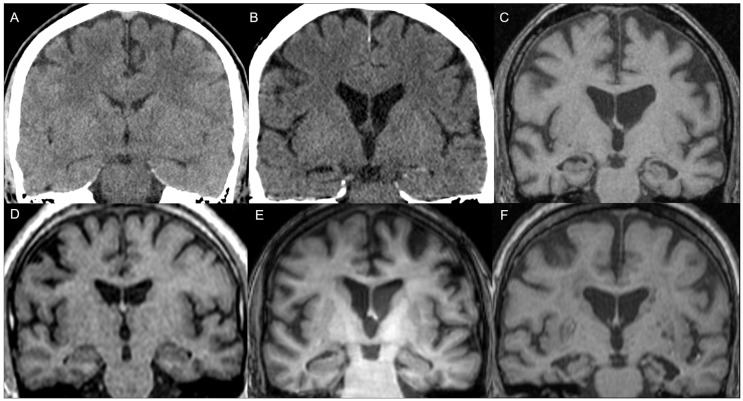
Examples of Medial Temporal Lobe Atrophy (MTA) scores in the sample. Bilateral MTA 0 in a 28-year-old patient who presented with auditive hallucinations on CT (**A**) and MRI (**D**); right MTA 1 and left MTA 0 on CT in a 40 year-old patient who presented with auditive and visual hallucinations (**B**) and in a 48-year-old patient who presented with auditive hallucinations on MRI (**E**); left MTA 2 and right MTA 1 on MRI in a 65-year-old patient who presented with auditive and visual hallucinations (**C**); right MTA 2 and left MTA 3 on MRI in a 77-year-old patient who presented with auditive hallucinations (**F**).

In the case of global MTA (average of right and left MTA), altered (MTA > 0) values were found in a total of 40 patients (38.1%), of which 38 patients (36.2%) were abnormal considering the age range. Fisher’s exact test showed a statistically significant association, with a higher presence of abnormal MTA for age in patients aged 50 years or older. The distribution of the overall MTA score by age range cut-offs is shown in Table 6 (and Supplementary Figure S4). Figure 3 shows illustrative examples of different MTA scores in patients from the sample.

#### 3.2.3. Non-Specific White Matter Lesions

**Figure 4:**
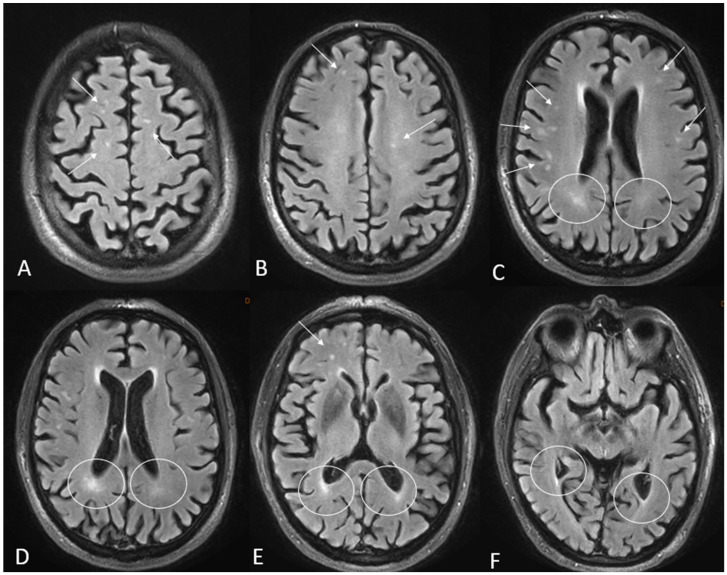
Illustrative examples of non-specific white matter lesions according to the Fazekas scale in two patients aged 53 and 77 years old who presented with first-episode auditive and visual hallucinations, respectively. (**A**–**F**) Axial T2 FLAIR sequences. Hyperintense T2 FLAIR foci can be observed in the deep white matter (arrows) and in the periventricular white matter (circles).

Within the group of patients with available MRI (n = 25), non-specific alterations of the white matter were found in 12 cases (48%); in 1 of these cases (4.3% of the total), the Fazekas scale indicated a number of lesions higher than expected considering age. The bivariate analysis according to the cut-off point of 70 years was not statistically significant, probably due to the low sample size (Table 7). Figure 4 shows examples of different Fazekas scale scores in the sample.

### 3.3. Specific Structural Brain Lesions

**Figure 5:**
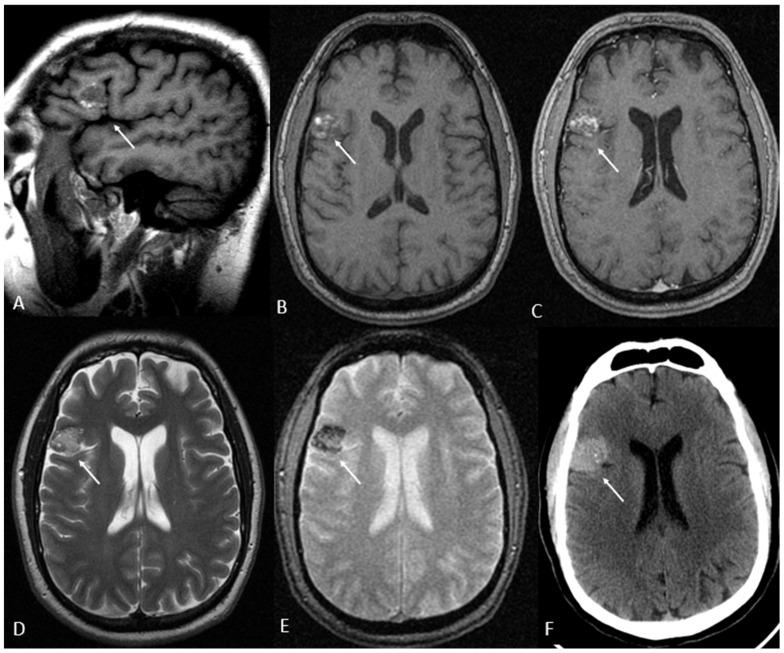
Right frontal opercular cavernoma on magnetic resonance imaging (MRI) (**A**–**E**) and computed tomography (CT) (**F**). T1-weighted sequences without intravenous contrast in sagittal (A) and axial planes (**B**), axial post-contrast T1-weighted (**C**), T2-weighted (**D**) and T2 GRE (**E**) images. There is an ovoid-like lesion in the right frontal opercular gyri containing hyperintense T1 and T2 areas, peripheral hypointense T2 rim, blooming artifact in T2 GRE and slight enhancement after intravenous contrast administration. In axial CT (**F**), the lesion corresponded to a hyperdense image with small punctiform calcifications. These findings are consistent with an opercular cavernoma (arrows). Note that no signs of acute complications were observed.

In one patient (1% of the total sample) who underwent both CT and MRI, a specific structural lesion was identified, which was a right frontal opercular cavernoma. The patient was a 38-year-old male with a history of anxiolytic use after a crash accident. He was brought to our hospital by the police after being arrested for breaking into a nursery school. The patient presented with psychotic contact with high internal tension that occasionally erupted into crying. His behavioral/cognitive disorganization was striking. His speech was completely disjointed and incomprehensible, bordering on gibberish, with neologisms and dysnomia. He made delusional, disconnected and unstructured statements, but these revealed feelings of guilt and a demand for punishment. No obvious hallucinatory phenomena were observed, but they could not be ruled out. Figure 5 shows the main imaging findings for this case.

## 4. Discussion

The present study shows results of interest for both clinical research and practice. First, we found a clear predominance of CT use (sole imaging examination in 76.2% of cases) compared to MRI (sole imaging examination in 7.6%). Second, we found that the presence of *any* non-specific structural abnormalities for age is high (45.7%) in FEP patients: cortical atrophy in 32.4%, medial temporal atrophy in 36.2% and non-specific white matter lesions in 4.3%. Finally, only one case (1%) presented a specific structural lesion (non-complicated cavernoma) located in the right frontal operculum. These findings suggest that the use of CT does not seem to be routinely justified in patients with FEP, and the usefulness and systematic use of MRI may be controversial, since it is necessary to determine the role and potential change in clinical decision-making of the non-specific alterations found. However, this real-world study has several limitations that warrant further investigations, highlighting the lack of a control group and the thresholds to consider non-specific alterations as abnormal for age.

One of the most relevant findings worth discussing lies in the nature and potential relevance of non-specific brain abnormalities in FEP. Indeed, all but one of the detected alterations are related to age-related changes whose relationship with psychosis is questionable. However, considering the age thresholds described in previous studies on the different types of alterations explored (cortical atrophy, mesial temporal and non-specific white matter lesions), a significant number of patients in our sample presented findings not in accordance with age.

To put our findings into context, we conducted a non-systematic review of the literature examining previous research studies that reported brain abnormalities in patients with FEP. The results (19 studies) are summarized in Supplementary Table S1. Not surprisingly, a high heterogeneity in terms of study design, selection criteria, structural lesions considered and conclusions regarding the utility of neuroimaging in FEP was found. Notably, five studies concluded that there were structural lesions associated with FEP [19,20,36,37,38], while thirteen studies concluded that only incidental abnormalities were observed without a causal relationship with FEP [11,25,39,40,41,42,43,44,45,46,47,48,49]. One study was explicitly inconclusive [50].

Research on neuroanatomical structural alterations in FEP is profuse and dates back several decades [41]. Recently, Vieira et al. conducted a multi-centric meta-analysis to analyze structural MRI abnormalities using voxel-based morphometry in more than 500 FEP patients and controls [51]. They found a consistent pattern of fronto-temporal, insular and occipital abnormalities in FEP, associated with the severity of symptoms and disease duration. Furthermore, the identification of specific microstructural alterations, such as reductions in the superior thalamic nuclei (mediodorsal and pulvinar), highlights the possibility of early biomarkers that could inform early intervention strategies in disorders such as schizophrenia [36]. Although these studies are of undoubted research interest, they are far from clinical practice because they require the use of complex image processing methods, such as diffeomorphic image registration optimized by twisted lie algebra [51,52].

On the other hand, previous studies have focused on examining the findings of neuroimaging in FEP in clinical practice, similar to our approach. Most of these studies supported the lack of impact of imaging findings on treatment approaches and subsequent prognosis. For instance, Lubman et al. [11] showed that the rate and severity of imaging abnormalities in FEP increase with age, being equivalent after age adjustment and in controls. Forbes et al. [12] analyzed multiple findings on neuroimaging tests to conclude that these were generally not necessary. Khandanpour et al. [25] also subscribed that routine implementation would not be necessary because most lesions are incidental.

Importantly, authors like Dazzan et al. [45] noted that neuroimaging may be useful to better understand the underlying mechanisms of the disease. In this regard, some studies suggest that neuroimaging may be instrumental in identifying key variations in the development of FEP and its possible evolution, in line with a neurodevelopmentalist approach to the disease as highlighted by Bellani et al. [20], in which white matter hyperintensities and ventricular asymmetries could indicate an increased risk of schizophrenia.

Contrary to the previously cited studies, the recent meta-analysis by Blackman et al. [24] reported a 6% prevalence of clinically significant findings (e.g., empty sella turcica, cortical atrophy, multiple vascular alterations, white matter hyperintensities, cysts and tumors) that required a change in clinical management. In the sensitivity analyses, the exclusion of a specific study [53] resulted in a 2.1 relative risk of clinically relevant abnormalities with respect to controls. According to the authors, these findings support the routine use of MRI as part of the initial assessment in patients presenting with FEP. However, one should question how and to what extent findings such as white matter abnormalities imply a clinical change in the management of FEP patients.

In agreement with our literature review, Blackman et al. highlighted the methodological heterogeneity of the included studies. In several cases, it was not possible to obtain information on the duration of treatment with antipsychotics or on other psychiatric comorbidities, which could have led to overrepresentation. In addition, many studies date from the 1990s and 2000s, when CT and MRI resolutions were lower, suggesting that some structural alterations might not have been correctly typed or overlooked. Notably, their meta-regression analysis found no association between the prevalence of clinically relevant abnormalities and publication year.

Remarkably, a possible explanation for the presence of structural abnormalities not justified by age in some younger patients may lie in the influence of illicit substances. There is growing evidence that methamphetamines, cannabis and ketamine can not only increase the risk of psychosis onset but may also accelerate cortical and hippocampal atrophy through neurotoxic effects [54,55]. Cannabis, in particular, has been consistently associated with a higher risk of FEP in genetically vulnerable individuals or those exposed to heavy or early-onset use [56]. Although our study design did not include detailed substance use data, the role of these factors should be considered in future studies.

Although our study has some limitations, the findings do not support the systematic use of neuroimaging in clinical practice. Importantly, the decision to perform structural neuroimaging in FEP must weigh a number of factors, namely diagnostic yield, patient safety (especially regarding ionizing radiation), clinical impact and cost. Brain CT is widely accessible and rapid, but involves exposure to ionizing radiation, with estimated effective doses between 1.5 and 2.0 mSv [57]. The lifetime projected risk of developing cancer from a single head CT in adults has been recently estimated in 12,500 cases, based on 2023 data from CT usage in the United States [58]. By contrast, MRI avoids radiation exposure but is more expensive, less accessible in emergency settings and may be poorly tolerated by claustrophobic patients.

The key issue lies in assessing the pre-test probability that neuroimaging will provide relevant information for clinical decision-making, in proportion to the risks involved. Age plays a critical role in modulating this risk–benefit balance. In younger adults (the predominant demographic in FEP), the likelihood of identifying clinically relevant structural pathology is low, while radiation sensitivity is higher, making the overall benefit of CT less favorable. In contrast, in older patients (e.g., over 50 or 60 years), the diagnostic yield of neuroimaging increases, as new-onset psychosis may be secondary to vascular, neurodegenerative or other organic brain conditions. The age-related distribution of structural abnormalities in our study supports this observation.

In light of the above-mentioned considerations, a rational approach would prioritize MRI (when available) or CT in cases with atypical features, while avoiding routine scanning in younger individuals with otherwise typical FEP presentations and no red flags. This strategy may help reduce unnecessary hospital costs and minimize risks associated with ionizing radiation. In fact, current clinical guidelines recommend neuroimaging only when additional clinical indicators are present—such as focal neurological deficits, recent head trauma or suspected stroke, seizure, or intracranial pathology [46,59].

Finally, regarding the specific structural lesion identified in one patient in our sample, we have found anecdotal case reports with similar findings in the literature. For instance, Raj et al. [60] described the case of a 21-year-old woman who developed post-partum psychosis and was found to have a small right temporal cavernoma on MRI, but the association with psychosis is more than questionable, considering the widely known association of post-partum states and psychosis onset. The authors acknowledged this as an incidental finding, but still linked both findings in causality terms. Majarwitz et al. reported a levetiram-induced psychotic episode in a patient with cavernomatosis [61]. Finally, Pavesi et al. reported a psychotic episode following bleeding of a corpus callosum cavernoma that was resolved following surgical management [62]. These anecdotal cases, along with the one found in our study, do not clarify the potential link between cavernomas and psychosis, and in our opinion, should be considered as incidental findings, particularly in the absence of complications.

### Strengths and Limitations of Our Study and Future Perspectives

One of the main strengths of our study lies in the selection of FEP patients with restrictive criteria, ensuring that they were in their first known psychotic episode by using ICD-10 standard codes and ruling out prior psychotic episodes. Second, we ensured that all imaging studies were performed within a close range of the episode, eliminating potential biases that could be present in previous studies, as it is known that many patients with FEP undergo neuroimaging several months after discharge. Third, all imaging studies were reviewed by a radiologist with 5 years of experience in neuroimaging. Finally, this is a real-world study, which gives an actual view of how neuroimaging is applied in a clinical practice setting.

However, this study has some limitations. First, this is a retrospective study, so there may be confounding factors that have not been controlled for, which results in an important limitation for establishing cause–effect relationships. In addition, the sample size is relatively small, which reduces the robustness of the statistical analyses, especially considering that many of the variables explored correspond to four-category scales (GCA, MTA, Fazekas). Third, no control group was used; thus, we relegated the reference standards of “normality” to age cut-offs previously described in the literature. In this regard, we observed a high variability in the described age thresholds for the three scales applied, and the ones chosen for this study could introduce misclassification bias. This reinforces the need for standardizing the currently applied neuroimaging scales for clinical practice, an issue that requires appropriate validation in future studies.

Due to the limitations of the present study, prospective studies with larger samples and with a well-defined control group would be advisable. Although our results do not support the routine implementation of neuroimaging in FEP, the use of advanced neuroimaging techniques such as diffusion tensor imaging on MRI may improve the clinical utility of neuroimaging tests in this context. Moreover, functional-focused examinations such as single-photon emission CT (SPECT) or standardized Low-Resolution Brain Electromagnetic Tomography (sLORETA) are increasingly contributing to advances in the current knowledge on psychosis and FEP and could offer reductions in costs compared to other neuroimaging modalities [63,64]. These examinations capture dynamic brain activity patterns, which may reflect altered connectivity or baseline dysfunctions not visible on structural scans. However, these methods are often less specific for diagnostic decision-making, may require advanced post-processing, and are not always standardized in clinical psychiatry. Thus, combining both functional and structural imaging (e.g., EEG, functional MRI and EEG-sLORETA) could help determine the most appropriate imaging assessment tools and the contextual relevance of imaging abnormalities in these patients.

In sum, our results point to the need for a clearer definition of the criteria for the indication of neuroimaging in psychiatric practice and clinical guidelines. Should clinical guidelines evolve to recommend structural neuroimaging in specific psychiatric contexts—such as FEP with atypical features or neurological red flags—we believe that both public and private insurance providers should consider coverage, ensuring equitable access and cost-effectiveness.

## 5. Conclusions

Neuroimaging tests used in clinical practice usually show non-specific alterations in patients with FEP, and the percentage of specific structural lesions is very low. The main non-specific alterations found correspond to global cortical and Medial Temporal Lobe Atrophy, which have been found in a higher percentage of cases than expected according to the thresholds previously described in the literature.

In addition, we found a predominant use of CT scans that does not seem justified, considering the absence of radiological abnormalities that modify the clinical approach to these patients and the risks associated with ionizing radiation. These findings support the need to rethink the rational use of neuroimaging tests in this clinical context, considering risk–benefit, cost efficiency and safety factors. Our results contribute to the existing real-world evidence on structural neuroimaging abnormalities in patients with FEP but should be validated by prospective studies, ideally multicentric, including a control group.

## Figures and Tables

**Table 1:** Characteristics of the Global Cortical Atrophy scale [27]. The reference thresholds are based on the studies of Ferreira et al. (2015) [28] and Rhodius-Meester et al. (2017) [29].

Score	Definition	Radiological Semiology	Threshold
0	Absent atrophy	No alterations	Normal at any age
1	Mild atrophy	Widening of cortical grooves	Indicates atrophy if <65 years
2	Moderate atrophy	Loss of volume of the gyri and convolutions	Pathological at any age
3	Severe atrophy (end-stage)	“Knife blade” atrophy	Pathological at any age

**Table 2:** Characteristics and assessment of the Medial Temporal Lobe Atrophy scale [30]. ↑ = Increase, ↓ = Decrease.

Score	Choroidal Fissure Width	Width of the Temporal Horn	Hippocampal Height
0	Normal	Normal	Normal
1	↑	Normal	Normal
2	↑↑	↑	↓
3	↑↑↑	↑↑	↓↓
4	↑↑↑	↑↑↑	↓↓↓

**Table 3:** Characteristics of the Fazekas scale [33,34]. The thresholds are based on the considerations of Wahlund et al.’s study [35].

Score	Periventricular Lesions	Deep White Matter Lesions	Threshold
0	Absent	Absent or isolated	Normal at any age
1	Layer or fine line	Multiple punctate foci	Normal at any age
2	Hyperintensities extending up to 10 mm from the ventricles	Partial confluence of hyperintensities	Pathological up to 70 years of age
3	Hyperintensities extending more than 10 mm from the ventricles.	Large confluent areas of hyperintensities	Pathological at any age

**Table 4:** Characteristics and main results in the study sample. GCA, Global Cortical Atrophy. MTA, Medial Temporal Lobe Atrophy. Quantitative variables are expressed as mean and standard deviation (X ± S.D.). Qualitative variables are expressed as absolute and relative frequencies (N [%]). ^ The Shapiro–Wilk test showed that age does not follow a normal distribution under the conventional significance threshold (*p* = 0.001); the median and interquartile range is 36 (28–53) years. * MRI was not performed, so the scale is not applicable. ** Including non-specific abnormalities for age (48 cases) and specific structural lesions (1 case).

Variable	Total Sample (n = 105) X ± S.D./N (%)
Age ^	39.5 ± 15.0
Gender (Female)	50 (47.6)
Neuroimaging test CT only MRI only CT and MRI	80 (76.2) 8 (7.6) 17 (16.2)
GCA scale score 0 1 2 3	71 (67.6) 27 (25.7) 6 (5.7) 1 (1)
MTA scale score, right 0 1 2 3 4	63 (60.0) 35 (33.3) 7 (6.7) 0 (0) 0 (0)
MTA scale score, left 0 1 2 3 4	65 (61.9) 32 (30.5) 7 (6.7) 1 (1) 0 (0)
Periventricular Fazekas scale score 0 1 2 3 Not assessed *	16 (15.2) 8 (7.6) 1 (1) 0 (0) 80 (76.2)
Fazekas score deep white matter 0 1 2 3 Not assessed *	14 (13.3) 10 (9.5) 1 (1) 0 (0) 80 (76.2)
Global Fazekas score 0 1 2 3 Not assessed *	13 (2.4) 10 (9.5) 2 (1.9) 0 (0) 80 (76.2)
Presence of any structural abnormality **	49 (46.7)

**Table 5:** Distribution of normal and abnormal Global Cortical Atrophy (GCA) scores by age cut-offs in the sample. ^a^ In this column, the percentage corresponds to the column counts. ^b^ In these columns, the percentages correspond to the row counts. * Significance value for Fisher’s exact test.

Age Range	Total Sample (N = 105) ^a^	GCA in Range (N = 71) ^b^	GCA Out of Range (N = 34) ^b^	Significance (*p*-Value) *
<65 years	99 (94.3)	71 (71.7)	28 (28.3)	<0.001
≥65 years old	6 (5.7)	0 (0)	6 (100)	

**Table 6:** Distribution of normal and abnormal Medial Temporal Lobe Atrophy (MTA) scores by age ranges in the sample. ^a^ In this column, the percentage corresponds to the column counts. ^b^ In these columns, the percentages correspond to the row counts. * Significance value for Fisher’s exact test.

Age Range	Total Sample (N = 105) ^a^	MTA in Range (N = 67) ^b^	MTA Out of Range (N = 38) ^b^	Significance (*p*-Value) *
<50 years	74 (70.5)	61 (86.5)	13 (13.5)	<0.001
50–64 years	25 (23.8)	4 (16)	21 (84)	
65–74 years	4 (3.8)	2 (50)	2 (50)	
≥75 years	2 (1.9)	0 (0)	2 (100)	

**Table 7:** Distribution of normal and abnormal Fazekas scores by age cut-offs. ^a^ In this column, the percentage corresponds to the column counts. **^b^** In these columns, the percentages correspond to the row counts. * *p*-value for Fisher’s exact test.

Age Range	Total Sample (N = 25) ^a^	Fazekas in Range (N = 23) ^b^	Fazekas Out of Range (N = 1) ^b^	Significance (*p*-Value) *
<70 years	23 (92)	22 (95.7)	1 (4.3)	1
≥70 years	2 (8)	2 (100)	0 (0)	

## Data Availability

All data from this study are available upon reasonable request to the corresponding author.

## Supplementary Materials

The following supporting information can be downloaded at https://www.mdpi.com/article/10.3390/jcm14144925/s1, Supplementary Figure S1. Age distribution in the sample; Supplementary Figure S2. Age distribution by sex in the sample; Supplementary Figure S3. Age distribution by neuroimaging examinations; Supplementary Figure S4. Distribution of in-range and out-of-range non-specific brain abnormalities in the sample. Supplementary Table S1. Studies included in the article related to the implementation of imaging studies. X + SD. Mean (X) and standard deviation (SD). NS. Not specified. ^ In some cases, the study included a larger sample size. The figure indicates the number of cases with first-episode psychosis (FEP) and neuroimaging examinations. ^^ Relative percentage relative to the entire sample size. * Overall conclusion against or for the causal association between neuroimaging abnormalities and first-episode psychosis (FEP).
