# Supplementary material for: The Value of Structural Neuroimaging in First-Episode Psychosis and the Prevalence of Imaging Abnormalities and Clinical Relevance: A Real-World Observational Study
Source: J Clin Med. 2025 Jul 11;14(14):4925. doi: 10.3390/jcm14144925 (PMC12294865; doi:10.3390/jcm14144925)
Supplement: Supplementary file 1 [file jcm-14-04925-s001.zip › Supplementary Files_revised/Supplementary Table S1.pdf]

Supplementary Table 1. Studies included in the article related to the implementation of imaging studies. X + SD. Mean (X) and standard deviation (SD). NS. Not specified. ^In some cases the study included a larger sample size. The figure indicates the number of cases with first-episode psychosis (FEP) and neuroimaging examinations. ^^ Relative percentage relative to the entire sample size. *Overall conclusion against or for the causal association between neuroimaging abnormalities and first-episode psychosis (FEP).

Study	Design	Control group	Neuroimaging examination	Sample size^	Age (years) (X ± SD / range)	Women (%)^^	Main findings	Conclusion*
Adams et al. (1996) [38]	Retrospective study	No	CT	98	13-19	38.7	11.0% of the neuroimaging screening tests were identified as positive. However, neither endocrine nor neuroimaging tests met the criteria for diagnostic utility.	Against
Andrea et al. (2019) [39]	Retrospective study	No	CT + MRI	443	15-24	47.3	Twenty-five (5.6%) of 443 subjects showed incidental findings unrelated to psychosis. The prevalence of positive neuroimaging findings was 0%, indicating no diagnostic yield from neuroimaging.	Against
Bain (1998) [40]	Retrospective study	No	CT	127	98 patients aged 17-30 years<br>23 patients aged 31-40 years<br>6 patients aged >40 years	19.69	Four incidental radiological abnormalities were found, none of which were causally related to psychosis	Against
Battaglia and Spector (1988) [41]	Prospective study	No	CT	45	17-54	33.33	Three individuals had incidental abnormalities, although none of them were considered to be implicated in psychosis	Against
Bellani et al. (2022) [19]	Retrospective study	Yes (healthy)	MRI	235	31.7 ± 10	42.98	Patients with FEP and chronic psychosis were 3-4 times more likely to show deep white matter hyperintensities than healthy controls.	For

Borgwardt et al. (2006) [42]	Prospective study	Yes (individuals at high risk of schizophrenia, depressive controls, healthy controls)	MRI	30	30.3 ± 6.9	26.67	The rate of radiological abnormalities in the FEP group was 40%, but only 2 of the 30 individuals had clinically relevant pathology	Against
Cho et al. (2019) [35]	Observational study	Yes (healthy controls)	MRI	73 (Control: 37)	22.4 ± 5.5	56.7	Reduced microstructural complexity in certain regions of the thalamus. Especially in the mediodorsal and pulvinar nuclei in patients with PEP.	For
Coentre et al. (2016) [43]	Retrospective study	No	CT + MRI	32 (29 CT, 1 MRI, 2 both)	29.6 ± 8.7	40.63	No patient presented brain abnormalities responsible for psychotic symptoms.<br>Thirty-seven percent had incidental findings (cerebral atrophy, arachnoid cysts, ventricular asymmetry/dilatation, plagiocephaly and calcification of the falx cerebri).	Against
Dazzan et al. (2004) [44]	Retrospective study	No	MRI	77	27.4 ± 8.1	40	FEP patients showed educed volume of gray matter, cerebral cortex and increased white matter in the left internal capsule.	Against
Falkenberg et al. (2017) (clinical) [45]	Retrospective study	Yes (healthy subjects)	MRI	241	14-56	32.78	Radiological abnormalities were found, but none required intervention nor were they related to psychosis	Against
Falkenberg et al. (2017) (research) [45]	Retrospective study	Yes (healthy subjects)	MRI	108	17-54	34.26	Radiological abnormalities were found, but none required intervention nor were they related to psychosis	Against

Gewirtz et al. (1994) [36]	Retrospective study	Yes (schizophrenia, schizoaffective disorder, bipolar disorder, psychotic depression)	CT	168	35 ± 12	52.98	One of the 168 individuals scanned (0.6%) had incidental findings that may have been causally related to psychosis. Cortical atrophy was present in 40% of patients. The frequency of atrophy increased with age, but did not differ between patients with controls	For
Khandanpour et al. (2013) [24]	Retrospective study	CT + MRI	No	316 (CT: 204; MRI: 112)	MRI: 59.3 (SD NS) CT: 66.8 (SD NS)	MRI: 37.7. TC: 58.8 RM: 37.7. TC: 58.8 RM: 37.7. TC: 58.8	Only 6 patients had lesions potentially responsible for FEP. Approximately 63% (MRI: 62.5%; CT: 65.2%) had incidental lesions. No significant differences between MRI and CT in the detection of organic diseases potentially responsible for the first psychotic episode ($p < 0.001$).	Against
Lieberman et al. (1993) [49]	Prospective study	Yes (heatlhy controls)	CT + MRI	66	24.3 ± 6	56	31% of FEP patients showed abnormalities in morphologic brain features (included areas: ventricles, frontal/parietal cortex and medial temporal lobe structures).	NS
Lubman et al. (2002) [11]	Retrospective study	Yes (healthy controls, chronic schizophrenia)	MRI	152	21.6 ± 3.5	31.6	22.2% of FEP patients were abnormal. 8.5% of patients required referral. Only in 3 patients findings (possible Huntington's disease, vascular lesion, arachnoid cyst) were relevant for disease management.	Against
McKay et al. (2006) [46]	Retrospective study	No	CT + MRI	52	15-26	NS	Only 4 cases (7.7%) had any abnormality, and only 2 cases (3.8%) required referral.	Against
Miller et al. (1991) [37]	Prospective study	Yes (healthy controls)	CT + MRI	24	60.1 ± 10.1	58.3	Patients with late-onset psychosis often have structural brain abnormalities and cognitive deficits, suggesting that structural brain damage is commonly associated with the late onset of psychosis	For

Robert Williams et al. (2014) [47]	Retrospective study	No	CT + MRI	115 (CT: 93; MRI: 14; CT+MRI: 8)	12-30	NS	Six incidental findiings (arachnoid cyst, aneurysm, mild cerebral atrophy, temporal angioma, calcification, mild cerebral volume loss). None of these findings were considered to be causal or of etiologic significance related to the presenting psychiatric symptoms	Against
Strahl et al. (2010) [48]	Retrospective study	No	CT	237	16-72	28.3	None of the CT scans revealed brain lesions that could be the cause or a contributing factor to the patient's psychosis. 17.6% of patients had findings that did not affect their clinical management.	Against
Watson et al. (2011) [18]	Prospective study	Yes (healthy controls)	MRI	49 (FEP in schizophrenia: 25; FEP in bipolar disorder: 24)	FEP in schizophrenia: 28.8 $\pm$ 9 FEP in bipolar disorder: 36 $\pm$ 10	FEP in schizophrenia: 24 FEP in bipolar disorder: 50	Relationship between change in hippocampal and amygdala volume correlated with severity of psychosis symptomatology since FEP.	For

5
