# Supplementary figures and images for: The Value of Structural Neuroimaging in First-Episode Psychosis and the Prevalence of Imaging Abnormalities and Clinical Relevance: A Real-World Observational Study
Source: J Clin Med. 2025 Jul 11;14(14):4925. doi: 10.3390/jcm14144925 (PMC12294865; doi:10.3390/jcm14144925)

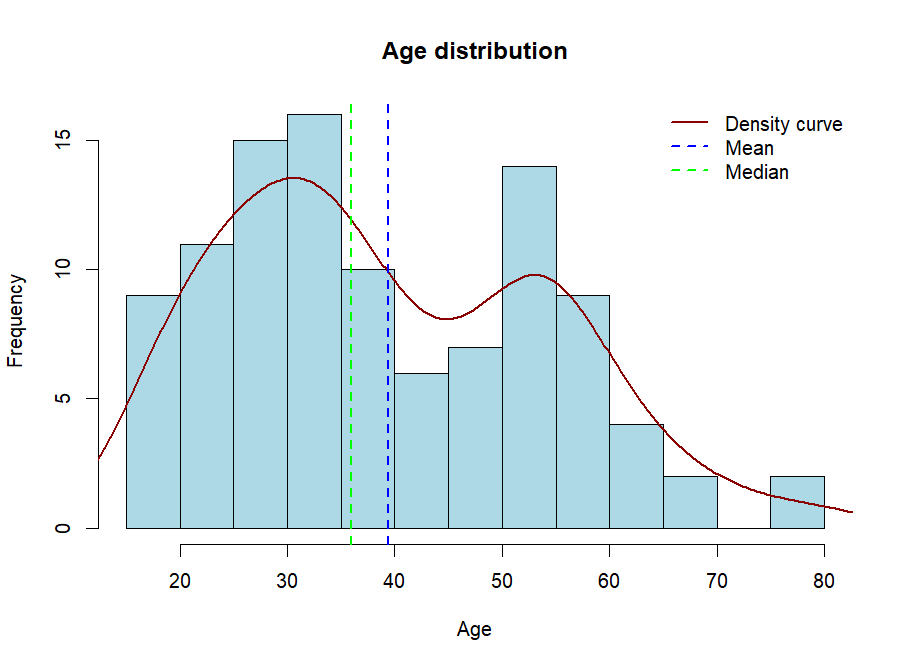

Supplement: Supplementary file 1 [file jcm-14-04925-s001.zip › Supplementary Files_revised/Supplementary Figure S1.png]

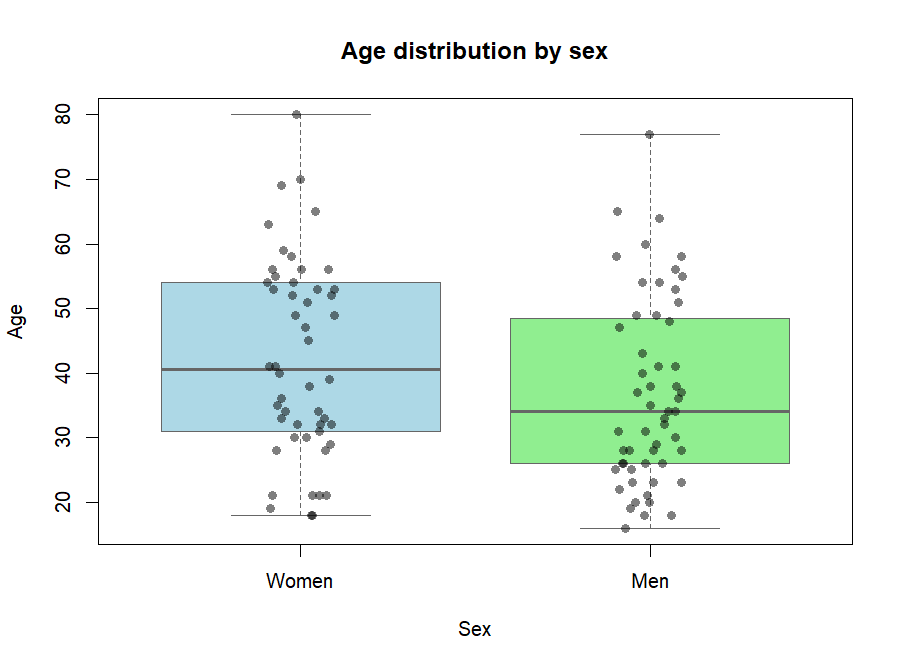

Supplement: Supplementary file 1 [file jcm-14-04925-s001.zip › Supplementary Files_revised/Supplementary Figure S2.png]

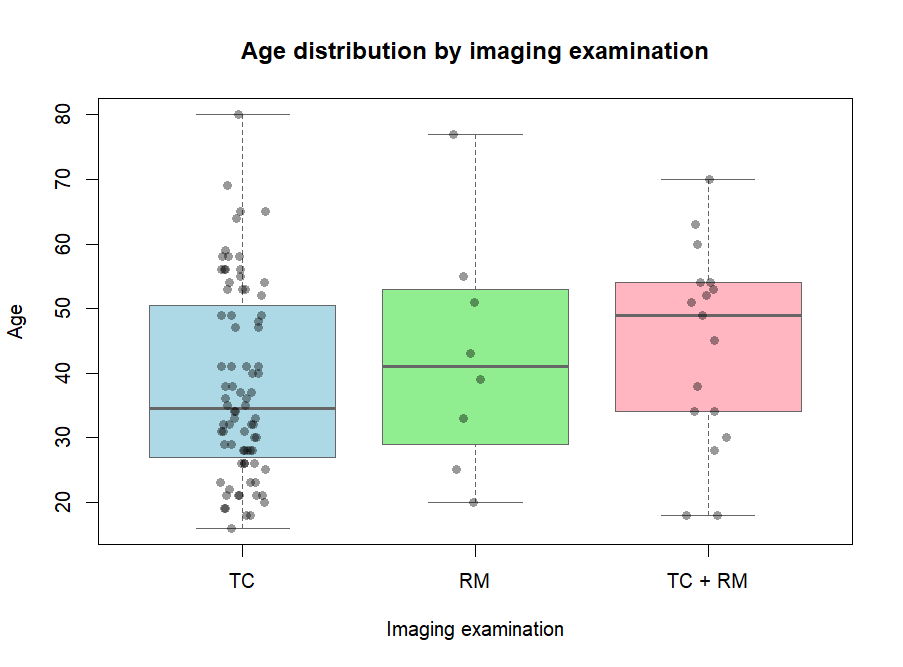

Supplement: Supplementary file 1 [file jcm-14-04925-s001.zip › Supplementary Files_revised/Supplementary Figure S3.png]

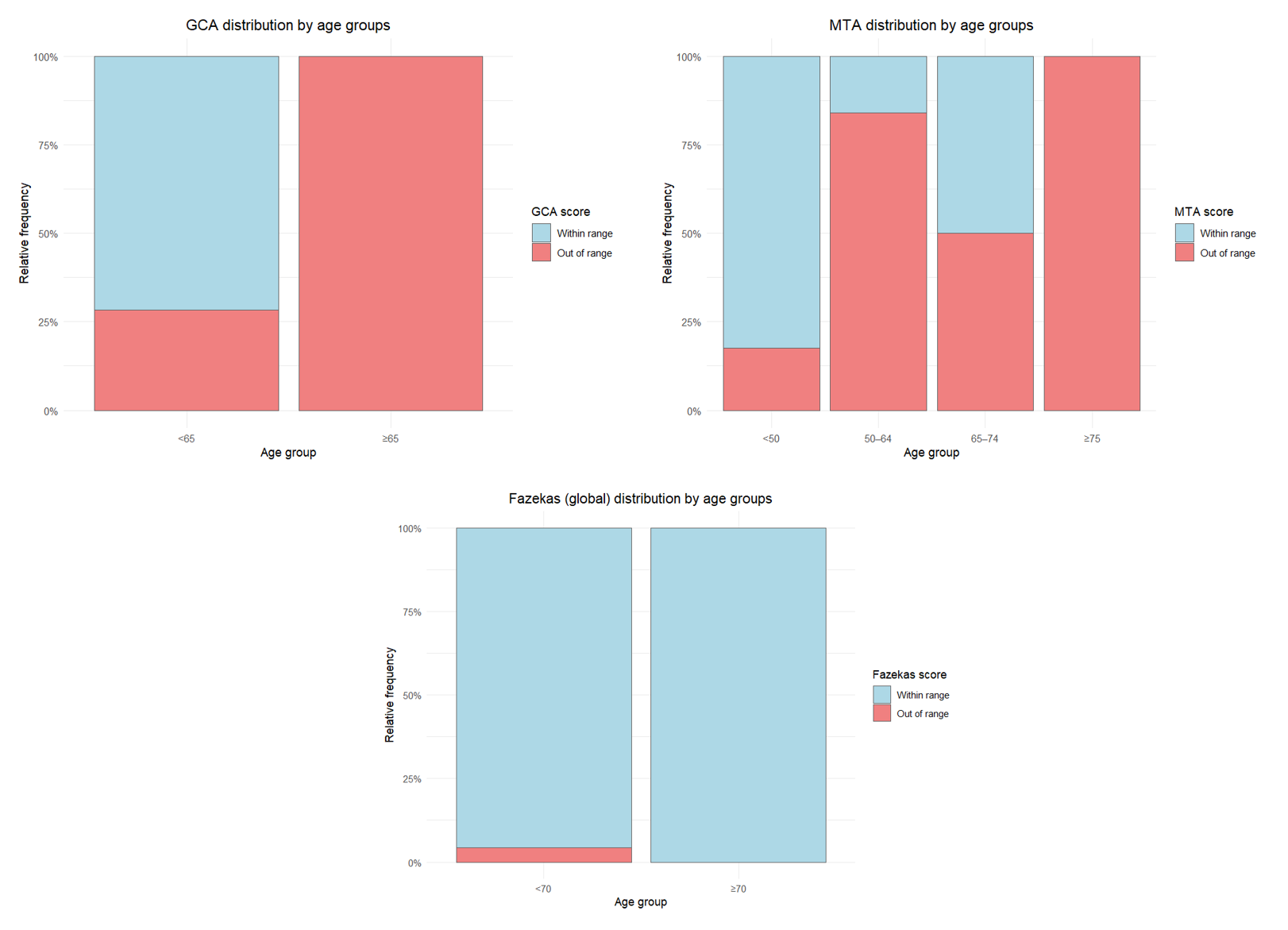

Supplement: Supplementary file 1 [file jcm-14-04925-s001.zip › Supplementary Files_revised/Supplementary Figure S4.png]
